# Faecal carriage of enterococci harbouring oxazolidinone resistance genes among healthy humans in the community in Switzerland

**DOI:** 10.1093/jac/dkac260

**Published:** 2022-08-16

**Authors:** Magdalena Nüesch-Inderbinen, Michael Biggel, Katrin Zurfluh, Andrea Treier, Roger Stephan

**Affiliations:** Institute for Food Safety and Hygiene, Vetsuisse Faculty, University of Zurich, 272 Winterthurerstrasse, 8057 Zurich, Switzerland; Institute for Food Safety and Hygiene, Vetsuisse Faculty, University of Zurich, 272 Winterthurerstrasse, 8057 Zurich, Switzerland; Institute for Food Safety and Hygiene, Vetsuisse Faculty, University of Zurich, 272 Winterthurerstrasse, 8057 Zurich, Switzerland; Institute for Food Safety and Hygiene, Vetsuisse Faculty, University of Zurich, 272 Winterthurerstrasse, 8057 Zurich, Switzerland; Institute for Food Safety and Hygiene, Vetsuisse Faculty, University of Zurich, 272 Winterthurerstrasse, 8057 Zurich, Switzerland

## Abstract

**Objectives:**

This study aimed to investigate the faecal carriage of enterococci harbouring oxazolidinone resistance genes among healthy humans in Switzerland and to genetically characterize the isolates.

**Methods:**

A total of 399 stool samples from healthy individuals employed in different food-processing plants were cultured on a selective medium containing 10 mg/L florfenicol. Resulting enterococci were screened by PCR for the presence of *cfr*, *optrA* and *poxtA*. A hybrid approach combining short-read and long-read WGS was used to analyse the genetic context of the *cfr*, *optrA* and *poxtA* genes.

**Results:**

*Enterococcus faecalis* (*n *= 6), *Enterococcus faecium* (*n *= 6), *Enterococcus gallinarum* (*n *= 1) and *Enterococcus hirae* (*n *= 2) were detected in 15/399 (3.8%) of the faecal samples. They carried *cfr *+ *poxtA*, *optrA*, *optrA *+ *poxtA* or *poxtA*. Four *E. faecalis* harbouring *optrA* and one *E. faecium* carrying *poxtA* were resistant to linezolid (8 mg/L). In most *optrA*-positive isolates, the genetic environments of *optrA* were highly variable, but often resembled previously described platforms. In most *poxtA*-positive isolates, the *poxtA* gene was flanked on both sides by IS*1216E* elements and located on medium-sized plasmids.

**Conclusions:**

Faecal carriage of *Enterococcus* spp. harbouring *cfr*, *optrA* and *poxtA* in healthy humans associated with the food-production industry demonstrates the possibility of spread of oxazolidinone resistance genes into the community. Given the importance of linezolid as a last-resort antibiotic for the treatment of serious infections caused by Gram-positive pathogens, the detection of the oxazolidinone resistance determinants in enterococci from healthy humans is of concern for public health.

## Introduction

The oxazolidinone antibiotic linezolid is one of the most important treatment options for severe infections caused by Gram-positive pathogens. In enterococci, linezolid resistance mechanisms include mutations in domain V of the 23S rRNA binding site and the acquisition of the transferable genes *cfr*, *optrA* or *poxtA*.^1^

Although linezolid-resistant enterococci (LRE) are reported globally at very low percentages (<1%),^2,3^ there remains cause for concern in view of possible horizontal gene transfer and dissemination of LRE within hospital environments.

Beyond the clinical setting, LRE have been detected throughout the agricultural sector, where they are co-selected by the use of florfenicol, a fluorinated derivative of chloramphenicol.^4^ Notably, *optrA* has been found frequently in isolates of animal origin, suggesting the possibility of foodborne transmission of this resistance determinant to humans.^5^

With currently few reports on human colonization with *optrA*-carrying enterococci,^6,7^ data on faecal carriage of enterococci harbouring oxazolidinone resistance genes in healthy humans remain scarce. Therefore, this study was designed to: (i) assess faecal carriage of enterococci harbouring oxazolidinone resistance genes among healthy individuals in Switzerland; (ii) characterize the isolates; and (iii) investigate the genetic context of the linezolid resistance determinants.

## Materials and methods

### Sampling and identification of enterococci harbouring oxazolidinone resistance genes

A total of 399 stool samples were obtained during September 2021 by the National Centre for Enteropathogenic Bacteria and Listeria (NENT) during a yearly routine *Salmonellae* screening of employees of a large food-processing company. This company consists of 10 countrywide processing plants and employs people from the surrounding urban communities. The study was approved by the local ethics committee of Zürich, Switzerland (BASEC-Nr.Req-2016-00374) and did not require participants’ consent.

Samples were processed and the presence of *cfr*-like genes, *optrA* and *poxtA* in enterococcal isolates was established by singleplex PCR as described previously.^8^

### Antimicrobial susceptibility testing

MICs of linezolid and chloramphenicol were determined using Etest (bioMérieux, Marcy-l’Étoile, France). Results were interpreted using the 2022 CLSI enterococci susceptibility breakpoints for broth microdilution.^9^

### WGS and genome analysis

Whole genomes were determined using short-read sequencing (Illumina MiniSeq, Illumina, San Diego, CA, USA). Isolates for which the genetic environment of *cfr*, *optrA* or *poxtA* could not be resolved from short-read data were additionally long-read sequenced on a MinION Mk1B device (Oxford Nanopore Technologies, Oxford, UK). Bacterial DNA extraction and sequencing, read assembly and *in silico* analyses are detailed in the Supplementary Materials and methods (available as Supplementary data at *JAC* Online).

### Nucleotide accession numbers

Sequencing data and genome assemblies are available under BioProject no. PRJNA783264. Assembly accession numbers are listed in Table S1 (available as Supplementary data at *JAC* Online).

## Results

### Isolation of enterococci harbouring oxazolidinone resistance determinants

Overall, 15 enterococci harbouring oxazolidinone resistance genes were retrieved from 399 samples, corresponding to a faecal carriage rate of 3.8%. A total of nine isolates harboured *optrA* alone (*n *= 6) or in combination with *poxtA* (*n *= 3), corresponding to an overall faecal carriage rate of *optrA*-positive enterococci of 2.3%. Nine isolates carried *poxtA* alone or in combination with *cfr* (*n *= 1) or with *optrA* (*n *= 3), corresponding to an overall faecal carriage rate of *poxtA*-positive enterococci of 2.3%.

### Antimicrobial susceptibility of the enterococcal isolates

As shown in Table S1, 5 (5/15, 33%) of the isolates were resistant to linezolid and 10 (10/15, 67%) were resistant to chloramphenicol.

### Genotyping of Enterococcus faecalis and Enterococcus faecium

MLST analysis of *E. faecalis* identified six different STs, including ST16, 32, 40, 207, 283 and 1008. *E. faecium* isolates were assigned to six STs (ST29, 104, ST108, 153, 272 and 1767). goeBURST analysis grouped all available STs into clonal complexes (CCs), which are listed in Table S1.

### Identification of cfr, optrA and poxtA variants

For the *optrA*-harbouring enterococci, nucleotide sequences were compared with the WT *optrA*_E349_ (GenBank accession number KP399637)^10^ and variants were defined based on alterations in the deduced amino acid sequences. A total of seven OptrA variants (including the WT) were identified (Table S1). They corresponded to OptrA_E349_, the EDM variant,^6^ identical at nucleotide level to that from *E. faecalis* E016 (GenBank accession no. KT862781),^11^ the EDD variant,^6^ corresponding to the gene from *E. faecalis* G20 (GenBank accession no. KT862784),^11^ the DP_2 variant,^1^ identical to that from *E. faecalis* 10-2-2 (GenBank accession no. KT862775),^11^ and the KLDP variant.^6^ Two novel OptrA variants, EDD_2 and EYNKWKVDASKELYNKQLEIG, respectively, were identified. The OptrA variants and their amino acid substitutions are listed in Table S2.

WGS analysis identified the WT *poxtA* gene, corresponding to that from *Staphylococcus aureus* AOUC-0915 (GenBank accession no. MF095097),^12^ and the *poxtA2* variant, identical to that from *Enterococcus gallinarum* Eg-IV02 (GenBank accession no. NG_076660).^13^ See Table S1.

### Genetic environment of optrA variants

**Figure 1.**
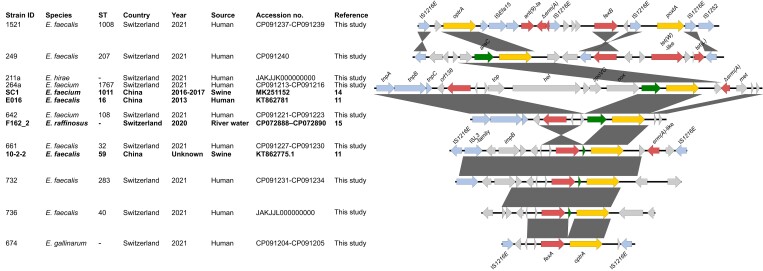
Genetic environments of *optrA* in enterococci from healthy humans. Strains with identical *optrA* platforms described previously are indicated in bold. Grey shades between sequences indicate homologous regions (100% sequence identity). Antimicrobial resistance genes are coloured in yellow (*optrA*, *poxtA*) or red and transposable elements in blue. The figure was generated using Easyfig 2.1 available at http://easyfig.sourceforge.net/. This figure appears in colour in the online version of *JAC* and in black and white in the print version of *JAC*.

As shown in Figure 1, the genetic environments of *optrA* were highly variable in the different isolates; however, they often resembled those described previously: in *Enterococcus hirae* 211a and *E. faecium* 264a, *optrA* was found on a shared platform that was associated with Tn*558* (*tnpA-tnpB*-*tnpC-orf138*-*fexA*) integrated into the chromosomal *radC* gene, as found in swine and human isolates elsewhere.^11,14^ See Figure 1. Similarly, in *E. faecium* 642, the *optrA*-containing platform consisted of the Tn*558*-associated genes and the *araC-optrA* module and was integrated at the *radC* site as described for aquatic *Enterococcus raffinosus*.^15^ See Figure 1.

*E. faecalis* 661 harboured an *impB-fexA-optrA* segment on a 25 kb plasmid previously described in porcine *E. faecalis*.^11^ See Figure 1. A similar environment was identified in the 38 kb plasmid of *E. faecalis* 732, differing, however, in the genes located downstream of *optrA* (Figure 1).

### Genetic environment of poxtA variants

**Figure 2.**
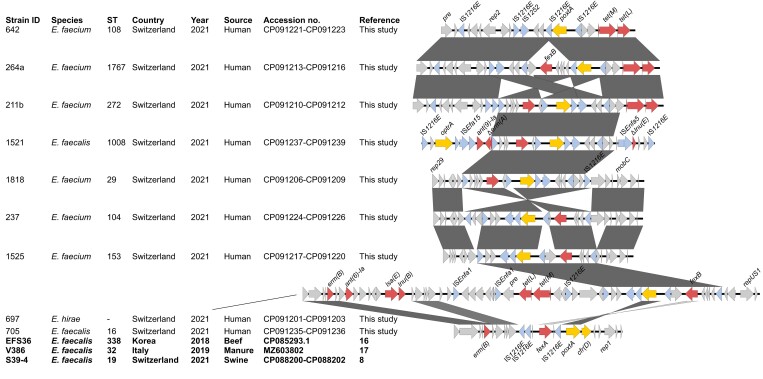
Genetic environments of *poxtA* in enterococci from healthy humans. Strains with identical *poxtA* platforms described previously are indicated in bold. Grey shades between sequences indicate homologous regions. Antimicrobial resistance genes are coloured in yellow [*optrA*, *poxtA*, *cfr*(D)] or red and insertion elements in blue. The figure was generated using Easyfig 2.1 available at http://easyfig.sourceforge.net/. This figure appears in colour in the online version of *JAC* and in black and white in the print version of *JAC*.

In most *poxtA-*positive isolates, *poxtA* was flanked on both sides by IS*1216E* elements located near *fexB* (Figure 2). Except for *E. faecalis* 1521, all isolates harboured *poxtA* on medium-sized plasmids. The plasmids in *E. faecium* 211b, 264a and 642 were structurally similar, as were the plasmids in *E. faecium* 237, 1525 and 1818 (Figure 2). Exceptionally, the 19 kb plasmid from *E. faecalis* 705 harboured the *poxtA2* allele, flanked upstream by an IS*1216E-fexA-*IS*1216E* segment and downstream by the *cfr*(D) gene, identical to *poxtA2* environments in food, swine and environmental isolates.^8,16,17^ See Figure 2.

## Discussion

In this study we found a faecal carriage rate of *optrA*-positive enterococci of 2.3%, which is lower than the 3.5% reported in a comparable study in healthy humans in China.^6^ Notably, there is a lack of comparative data on the presence of *poxtA*-harbouring enterococci in healthy humans. However, one of the few available studies reported a prevalence of 1%.^18^ With a prevalence of *poxtA* of 2.3%, our data suggest that *optrA* and *poxtA* occur with equal frequency among enterococci in healthy humans living in the community. However, it must be noted that these findings apply primarily to individuals with occupational exposure to food and may therefore not be directly generalized to the entire community.

Phenotypic resistance to linezolid was observed for *E. faecalis* carrying OptrA_E349_, the DP_2 variant and the KLDP variant. In all cases, the resistance determinants were plasmid encoded and represented the simplest versions of *optrA* contexts described in this study. A comparison with other isolates harbouring identical *optrA* platforms showed that resistance levels to linezolid may vary. For example, in isolate 661, the *optrA* region was identical to that found in linezolid-susceptible porcine *E. faecalis* 10-2-2 from China.^11^ Similarly, linezolid resistance was associated with *E. faecium* 211b containing a *poxtA* environment identical to that found in susceptible *E. faecium* 1521 from this study. Thus, it is interesting that linezolid MIC levels may vary substantially despite a common resistance determinant within identical genetic environments. Further, the finding of *optrA* and *poxtA* in genetic contexts identical to those found in human and animal enterococci in different geographical regions suggests the occurrence of independent genetic events linked to IS1216E and Tn*558*-like elements, and to plasmids belonging to repA_N or other replicon families.

Likewise, various *E. faecalis* and *E. faecium* STs identified in this study have been described in clinical and animal settings worldwide. For instance, *optrA*-positive *E. faecalis* ST16 (CC16) has been identified among clinical isolates in China, Greece and Denmark, and in pigs and poultry in Korea.^1,19^ Further, *optrA*-positive *E. faecalis* ST32 (CC4) and *optrA*-positive *E. faecium* ST29 (CC17) have been described in pigs and poultry in Korea and China.^19^ Recently, *optrA*-positive *E. faecalis* ST40 (CC40) and *optrA *+ *poxtA*-positive *E. faecalis* ST1008 were detected in raw meat-based pet food in Portugal.^20^ The occurrence of these STs in healthy individuals highlights their potential to spread between animals and humans, with implications for public health.

### Conclusions

This study provides novel insight into the role of the healthy human gut as a reservoir of *cfr*-, *optrA*- and *poxtA*-positive enterococci. The occurrence of enterococci harbouring clinically relevant oxazolidinone resistance determinants in genetic environments that have been described in clinical isolates as well as in livestock-associated settings worldwide is of epidemiological interest. Regular and updated information on the occurrence of oxazolidinone resistance genes in enterococcal isolates is essential to anticipate future trends in the prevalence and dissemination of *cfr*, *optrA* and *poxtA*.

## Supplementary Material

dkac260_Supplementary_Data
