# Supplementary material for: Faecal carriage of enterococci harbouring oxazolidinone resistance genes among healthy humans in the community in Switzerland
Source: J Antimicrob Chemother. 2022 Aug 16;77(10):2779–83. doi: 10.1093/jac/dkac260 (PMC9525073; doi:10.1093/jac/dkac260)
Supplement: dkac260_Supplementary_Data [file dkac260_supplementary_data.docx]

**Supplementary data**

**Supplementary Materials and methods**

Whole genomes were determined using short-read sequencing (Illumina MiniSeq, Illumina, San Diego, CA, USA). Isolates for which the genetic environment of *cfr*, *optrA*, or *poxtA* could not be resolved from short-read data were additionally long-read sequenced on a MinION Mk1B device (Oxford Nanopore Technologies, Oxford, UK).

Isolates were grown on sheep blood agar (Difco, Becton Dickinson, Allschwil, Switzerland), and genomic DNA was extracted using the DNeasy Blood & Tissue Kit (Qiagen, Hombrechtikon, Switzerland). For short-read sequencing, libraries were prepared using the Nextera DNA Flex Library Preparation Kit (Illumina, San Diego, CA, USA), and sequencing was performed on the Illumina MiniSeq platform with 2 × 150 bp paired-end chemistries. Illumina read adapters and low-quality bases were trimmed with TrimGalore v0.6.61 (<https://github.com/FelixKrueger/TrimGalore>) and quality assessed with FastQC v0.11.9 (https://www.bioinformatics.babraham.ac.uk/projects/fastqc/). For long-read sequencing, libraries were prepared using the SQK-LSK109 Ligation Sequencing Kit (Oxford Nanopore Technologies, Oxford, UK) and sequenced on a MinION Mk1B device using the FLO-MIN106 (R9) flow cell (Oxford Nanopore Technologies, Oxford, UK). Basecalling, demultiplexing, and barcode trimming was performed with guppy v4.2.2 (Oxford Nanopore Technologies, Oxford, UK) and quality assessed with LongQC v1.2.0.**^1^**

Short-read data assemblies were generated using SPAdes v.3.14.1**^2^** implemented in shovill v1.1.0.**^3^** Hybrid assemblies were generated with Unicycler v.0.4.8**^4^** using default settings. Genes were annotated using PGAP 2021-01-11.build5132**^5^** and isolates were typed in silico using mlst v.2.19.0*.***^6^** Clonal complexes (single linkage variants) were assigned using PHYLOVIZ 2.0 in combination with the goeBURST algorithm.**^7^** Clade affiliation of *E. faecium* was determined by phylogenetic clustering with 50 publicly available genomes for which clades were previously assigned.**^8^** Following core genome alignment using Snippy v.4.6.0 (https:// github.com/tseemann/snippy) with the chromosome of *E. faecium* SRR24 (GCF_009734005.1) as reference and masking of recombinant regions detected with Gubbins v.2.4.1,**^9^** clusters were defined from the SNP alignment using fastbaps v.1.0.5.**^10^** Antimicrobial resistance genes and plasmid replicons were identified using ABRicate 1.0.0**^11^** (70% coverage, 90% identity) in combination with the ResFinder**^12^** and PlasmidFinder databases,**^13^** respectively. Plasmid replicon families were determined based on conserved domains identified in replicon sequences using NCBI BLAST.**^14^** Allele numbers of *optrA* were assigned according to the scheme from Freitas et al.**^15^** Reads were also queried for mutations in the 23S rRNA genes associated with linezolid resistance (G2505A and G2576T) using LRE-finder 1.0.**^16^**

**Table S1.** Characteristics of 15 enterococci harbouring oxazolidinone resistance genes from healthy humans in Switzerland

|  |  |  |  | MIC (mg/L) | |  |  |  |  |
| --- | --- | --- | --- | --- | --- | --- | --- | --- | --- |
| Isolate ID | Species | MLST | CC (clade) | CL^a^ | LZ ^a^ | Oxazolidinone resistance determinants^b^ | Location of linezolid-resistance determinants (plasmid size/replicon type [group]) | Other antimicrobial resistance genes^c^ | WGS accession number |
| 1521 | *E. faecalis* | ST1008 | - (-) | 128 | 4 | *optrA* (EYNKWKVDASKELYNKQLEIG)*, *poxtA* | Chromosome | *ant(9)-Ia, cat, dfrG, erm*(B)*, fexB, lsa*(A)*, tet*(L)*, tet*(M) | CP091237-CP091239 |
| 705 | *E. faecalis* | ST16 | CC16 (-) | 128 | 2 | *cfr*(D), *poxtA2* | Plasmid (19 kb/rep1 [PriCT_1]) | *aac(6')-aph(2''), ant(6)-Ia, aph(3')-III, cat, erm*(B)*, fexA, lnu*(B)*, lsa*(A)*, lsa*(E)*, tet*(M) | CP091235-CP091236 |
| 249 | *E. faecalis* | ST207 | CC28 (-) | 24 | 4 | *optrA* (EDD_2)* | Chromosome | *fexB, lsa*(A)*, tet*(M)*, tet*(O/W/32/O) | CP091240 |
| 732 | *E. faecalis* | ST283 | CC25 (-) | 128 | 24 | *optrA* _E349_ | Plasmid (38 kb/repUS40 [Rep_3]) | *ant(6)-Ia, aph(3')-III, cat(pC221), erm*(B)*, fexA, lsa*(A)*, tet*(L)*, tet*(M) | CP091231-CP091234 |
| 661 | *E. faecalis* | ST32 | CC4 (-) | 256 | 8 | *optrA* (DP_2) | Plasmid (25 kb/rep9b [RepA_N]) | *aac(6')-aph(2''), ant(6)-Ia, ant(6)-Ia, aph(3')-III, cat, dfrG, erm*(A)*, erm*(B)*, fexA, lnu*(B)*, lsa*(A)*, lsa*(E)*, tet*(L)*, tet*(M) | CP091227-CP091230 |
| 736 | *E. faecalis* | ST40 | CC40 (-) | 32 | 16 | *optrA* _E349_ | Plasmid (38 kb/rep8b [RepA_N]) | *fexA, lsa*(A) | JAKJJL000000000 |
| 237 | *E. faecium* | ST104 | CC17 (A2) | 24 | 4 | *poxtA* | Plasmid (24 kb/rep29 [Rep_3]) | *aac(6')-Ii, fexB, msrC, tet*(L)*, tet*(M) | CP091224-CP091226 |
| 642 | *E. faecium* | ST108 | CC94 (B) | 48 | 4 | *optrA* (EDD), *poxtA* | *optrA*: Chromosome  *poxtA*: Plasmid (22 kb/rep2 [n.i.]) | *aac(6')-Ii, aac(6')-aph(2''), erm*(B)*, fexA, lnu*(B)*, lnu*(G*), lsa*(E)*, msrC, tet*(L)*, tet*(M)*, aph(3')-III* | CP091221-CP091223 |
| 1525 | *E. faecium* | ST153 | CC17 (A2) | 32 | 6 | *poxtA* | Plasmid (21 kb/rep29 [Rep_3]) | *aac(6')-Ii, ant(6)-Ia, fexB, lnu*(B)*, lsa*(E)*, tet*(L)*, tet*(M) | CP091217-CP091220 |
| 264a | *E. faecium* | ST1767 | CC17 (A2) | 32 | 3 | *optrA* (EDM), *poxtA* | *optrA*: Chromosome  *poxtA*: Plasmid (27 kb/rep2 [n.i.]) | *aac(6')-Ii, ant(6)-Ia, ant(6)-Ia, aph(3')-III, erm*(A)*, erm*(B)*, fexA, fexB, lnu*(B)*, lsa*(E)*, msrC*)*, tet*(L)*, tet*(M) | CP091213-CP091216 |
| 211b | *E. faecium* | ST272 | CC17 (A2) | 32 | 8 | *poxtA* | Plasmid (28 kb/rep2 [n.i.]) | *aac(6')-Ii, fexB, tet*(L)*, tet*(M) | CP091210-CP091212 |
| 1818 | *E. faecium* | ST29 | CC17 (A2) | 24 | 4 | *poxtA* | Plasmid (24 kb/rep29 [Rep_3]) | *aac(6')-Ii, fexB, msrC, tet*(L)*, tet*(S) | CP091206-CP091209 |
| 674 | *E. gallinarum* | - | - (-) | 256 | 8 | *optrA* (KLDP) | Plasmid (35 kb/repUS1 [n.i.]) | *VanC1XY, aac(6')-aph(2''), cat, erm*(B)*, fexA, lnu*(B)*, lsa*(E)*, str, tet*(L)*, tet*(M) | CP091204-CP091205 |
| 211a | *E. hirae* | - | - (-) | 12 | 3 | *optrA* (EDM) | Chromosome | *aac(6')-Iid, erm*(A)*, fexA* | JAKJJK000000000 |
| 697 | *E. hirae* | - | - (-) | 8 | 1.5 | *poxtA* | Plasmid (53 kb/repUS1 [n.i.]) | *aac(6')-Iid, ant(6)-Ia, erm*(B)*, fexB, lnu*(B)*, lsa*(E)*, tet*(L)*, tet*(M) | CP091201-CP091203 |

Clonal complex; CL, chloramphenicol; LZ, linezolid, MIC, minimal inhibitory concentration; MLST, multilocus sequence type; n.i., not identified; *, novel variant; -, not applicable.

^a^ Breakpoints for resistance were ≥32 mg/L for CL and ≥8mg/L for LZ, according to CLSI.**^17^** *E. faecalis* ATCC 29212 was used as a quality control strain (MIC of CL: 6 mg/L and MIC of LZ: 1.5 mg/L).

^b^ The *optrA* nomenclature corresponds to that of Schwarz et al.**^18^** The *optrA* _E349_ gene is identical to the wildtype (GenBank accession number KP399637)**^19^**. Amino acid changes corresponding to *optrA* gene mutations are indicated in brackets for each *optrA* variant. Amino acid substitutions and their positions are shown in Supplementary Table S2. Asterisks indicate novel variants.

^c^ Resistance genes located on plasmids harbouring oxazolidinone resistance genes are underlined.

**Table S2.** OptrA variants detected among nine enterococci from healthy humans in Switzerland

| OptrA variant | Amino acid substitution(s) and positions^a^ | *Enterococcus* spp. (no. isolates) | Reference |
| --- | --- | --- | --- |
| Wildtype OptrA_E349_ | none | *E. faecalis* (2) | 19 |
| DP_2 | Y176**D**, T481**P** | *E. faecalis* (1) | 18 |
| EDD | K3**E**, Y176**D**, G393**D** | *E. faecium* (1) | 20 |
| EDD_2 | K3**E**, G40**D**, G393**D** | *E. faecalis* (1) | This study |
| EDM | K3**E**, Y176**D**, I622**M** | *E. faecium* (1) *E. hirae* (1) | 20 |
| EYNKWKVDASKELYNKQLEIG | K3**E**, N12**Y**, G40**N**, N122**K**, Y135**W**, I287**K**, A350**V**, G393**D**, V395**A**, A396**S**, Q509**K,** Q541**E,** M552**L**, N560**Y**, K562**N**, Q565**K**, S614**Q**, I627**L**, D633**E**, N640**I,** R659**G** | *E. faecalis* (1) | This study |
| KLDP | T112**K**, S147**L**, Y176**D**, T481**P** | *E. gallinarum* (1) | 20 |

^a^ Substituted amino acids are shown in bold.

**References**

**1** Fukasawa Y, Ermini L, Wang H *et al.* LongQC: A quality control tool for third generation sequencing long read data. *G3: Genes, Genomes, Genetics* 2020; **10**:1193-6.

**2** Bankevich A, Nurk S, Antipov D *et al.* SPAdes: a new genome assembly algorithm and its applications to single-cell sequencing. *J Comput Biol* 2012; **19**:455-77.

**3** Seemann T. Shovill. https://githubcom/tseemann/shovill. 2019.

**4** Wick RR, Judd LM, Gorrie CL *et al.* Unicycler: resolving bacterial genome assemblies from short and long sequencing reads. *PLoS Comput Biol* 2017; **13**:e1005595.

**5** Tatusova T, DiCuccio M, Badretdin A *et al.* NCBI prokaryotic genome annotation pipeline. *Nucleic Acids Res* 2016; **44**:6614-24.

**6** Seemann T. mlst. https://github.com/tseemann/mlst. 2021;

**7** Nascimento M, Sousa A, Ramirez M *et al.* PHYLOViZ 2.0: providing scalable data integration and visualization for multiple phylogenetic inference methods. *Bioinformatics* 2017; **33**:128-9.

**8** Gouliouris T, Coll F, Ludden C *et al.* Quantifying acquisition and transmission of *Enterococcus faecium* using genomic surveillance. *Nature Microbiol* 2021; **6**:103-11.

**9** Croucher NJ, Page AJ, Connor TR *et al.* Rapid phylogenetic analysis of large samples of recombinant bacterial whole genome sequences using Gubbins. *Nucleic Acids Res* 2015; **43**:e15-e15.

**10** Tonkin-Hill G, Lees JA, Bentley SD *et al.* Fast hierarchical Bayesian analysis of population structure. *Nucleic Acids Research* 2019; **47**:5539-49.

**11** Seemann T. Abricate. https://github.com/tseemann/abricate. 2021.

**12** Bortolaia V, Kaas RS, Ruppe E *et al.* ResFinder 4.0 for predictions of phenotypes from genotypes. *J Antimicrob Chemother* 2020; **75**:3491-500.

**13** Carattoli A, Zankari E, García-Fernández A *et al.* In silico detection and typing of plasmids using PlasmidFinder and plasmid multilocus sequence typing. *Antimicrob Agents Chemother* 2014; **58**:3895-903.

**14** Madden T. The BLAST sequence analysis tool. *The NCBI handbook* 2002; **2**:425-36.

**15** Freitas AR, Tedim AP, Novais C *et al.* Comparative genomics of global *optrA*-carrying *Enterococcus faecalis* uncovers a common chromosomal hotspot for *optrA* acquisition within a diversity of core and accessory genomes. *Microb Genom* 2020; **6:** e000350.

16 Hasman H, Clausen PTLC, Kaya H *et al.* LRE-Finder, a web tool for detection of the 23S rRNA mutations and the *optrA*, *cfr*, *cfr*(B) and *poxtA* genes encoding linezolid resistance in enterococci from whole-genome sequences. *J Antimicrob Chemother* 2019; **74**:1473-6.

17 Clinical and Laboratory Standards Institute. *Performance standards for antimicrobial susceptibility testing: Thirty-second Edition M100.* CLSI, Wayne, PA, USA, 2022.

18 Schwarz S, Zhang W, Du XD *et al.* Mobile oxazolidinone resistance genes in Gram-positive and Gram-negative bacteria. *Clin Microbiol Rev* 2021; **34**:e0018820.

19 Wang Y, Lv Y, Cai J *et al.* A novel gene, *optrA*, that confers transferable resistance to oxazolidinones and phenicols and its presence in *Enterococcus faecalis* and *Enterococcus faecium* of human and animal origin. *J Antimicrob Chemother* 2015; **70**:2182-90.

20 Cai J, Schwarz S, Chi D *et al.* Faecal carriage of *optrA*-positive enterococci in asymptomatic healthy humans in Hangzhou, China. *Clin Microbiol Infect* 2019; **25**:630.e1-6.
